# Intra-Individual Variability of Error Awareness and Post-error Slowing in Three Different Age-Groups

**DOI:** 10.3389/fpsyg.2018.00902

**Published:** 2018-06-05

**Authors:** Fabio Masina, Elisa Di Rosa, Daniela Mapelli

**Affiliations:** ^1^Department of General Psychology, University of Padova, Padova, Italy; ^2^Department of Neuroscience, University of Padova, Padova, Italy; ^3^Human Inspired Technologies Research Center, University of Padova, Padova, Italy

**Keywords:** error awareness, post-error slowing, intra-individual variability, executive functions, performance monitoring, ex-Gaussian analysis

## Abstract

**Background:** Error awareness (EA) and post-error slowing (PES) are two crucial components of an adequate performance monitoring because, respectively, they allow being aware of an error and triggering performance adjustments following unexpected events.

**Objective:** The purpose of the present study was to investigate the ontogenetic trajectories of EA and PES, as well as to examine how EA and PES interact with each other.

**Methods:** The performance of three groups of participants (children, younger, and older adults) in a modified version of the Error Awareness task (EAT; Hester et al., 2005) was compared. In particular, in this study not only variations of the average performance were examined, but also intra-individual variability (IIV), considered in terms of variations of SD and ex-Gaussian parameters (*mu*, *sigma*, and *tau*).

**Results:** Two distinct ontogenetic trajectories of EA and PES were observed. Regarding EA, we observe a U-shaped curve that describes an increase of the process from childhood to early adulthood and a progressive reduction advancing age in late adulthood. Furthermore, a greater IIV in older adults indicated a susceptibility of EA to the aging process. The ontogenetic trajectory of PES seems substantially different from the trajectory that describes EA since in PES we do not observe age-related differences.

**Conclusion:** These results suggest that EA and PES are two independent processes. Furthermore, it appears that EA and PES are differently prone to short-term fluctuations in performance across the lifespan. While EA presents an increase in IIV in aging, PES seems to be immune to these changes.

## Introduction

The ability to monitor our performance, and moreover our errors, is essential in everyday life. In fact, an error not only represents a failure during performance but also a source of information about the necessity, direction, and magnitude of adjustments needed to prevent similar errors in future (Ullsperger et al., 2014). Therefore, only a correct performance monitoring allows triggering compensatory actions in order to support an efficient goal-directed behavior (Ullsperger and von Cramon, 2006).

Among the processes that constitute performance monitoring, the most representative and investigated phenomena are *post-error slowing* (PES) and *error awareness* (EA). Both these two phenomena have been widely studied in the literature, from several interesting points of view.

Post-error slowing is the motor slowing that usually occurs after errors, and was described for the first time in 1966 by Rabbitt, who reported significant slower reaction times (RTs) after erroneous responses than mean RTs of all correct responses (Rabbitt, 1966). From this first evidence, other studies have reported PES in different kinds of task, for instance Stroop, Simon, Flanker or categorization tasks (for a review, Danielmeier and Ullsperger, 2011). However, despite this large piece of knowledge, the functional role of PES is still debated. Specifically, two veins of research consider alternatively PES either an adaptive or a maladaptive phenomenon. On the one hand, the adaptive theories claim that PES contributes to improving ongoing behavior, involving both the perceptual and the motor system. For instance, Botvinick et al. (2001) suggest that PES might reflect the modification of the amount of sensory evidence required to initiate a motor response. On the other hand, the maladaptive theories propose that PES would be a detrimental consequence of an impaired processing after an error. In line with this idea, Notebaert et al. (2009) propose that PES would be caused by the relative infrequency of errors during a task, which in turn may cause attentional lapses. In fact, in their study, when the error rate increased, approaching the frequency of the correct responses, PES was reduced or absent (Notebaert et al., 2009).

Despite these two different theories, PES can be considered as an index of performance adjustments following unexpected events (Wessel and Aron, 2017).

However, contrary to EA, PES does not inform about the level of consciousness in error detection.

Error awareness is a metacognitive process that allows being aware of an error. Relatively more recent respect to the analysis of PES, Hester et al. (2005) introduced an interesting method to investigate EA, with the design of a specific task, i.e., the Error Awareness Task (EAT; Hester et al., 2005). From that first study, EA has been the object of several works (Shalgi et al., 2007; O’Connell et al., 2009; Harty et al., 2013, 2014), where different aspects have been explored: from its neural basis and electrophysiological markers, to its relation with other cognitive functions and environmental aspects. Specifically, it seems that EA strictly depends on both endogenous factors, such as attention or expertise, and exogenous factors, such as time pressure or ambiguity in task situations (Klein et al., 2013). Interestingly, other studies have shown EA can be significantly affected by different neurological and psychiatric diseases (Klein et al., 2013), and to decline in normal aging (Harty et al., 2013).

Nonetheless, despite these two phenomena have been largely studied, at least two questions appear to be still open in this literature: How do these two mechanisms develop across the lifespan? And how do they interact?

The changes of PES and EA across the lifespan seem topics scarcely explored in literature, and to the best of our knowledge, so far no study has investigated intra-individual variability (IIV) of PES and EA across different age-groups.

Moreover, the evidence about a possible correlation between PES and EA is not convergent.

In fact, several authors recently showed that PES is significantly modulated by EA since it is larger after an aware error than unaware (Nieuwenhuis et al., 2001; Hester et al., 2005; Wessel and Ullsperger, 2011). At the same time, other evidence seems to show the opposite, reporting significant PES also after unnoticed errors (Cohen, 2009).

To date, studies that have jointly investigated PES and EA are still few and this would explain the fact that it is not yet possible to clearly accept or exclude an association between the two processes.

With the goal to identify the ontogenetic trajectories of EA and PES, the present study reports new evidence from healthy subjects of different age-groups, where EA and PES have been analyzed in terms of both intra and inter-individual variability.

In fact, although the ontogenetic cognitive development has been mainly investigated through the comparison of participants’ average performance, only the study of IIV allows understanding another important aspect, such as the short-term changes in behavior. For the sake of clarity, with “intra-individual variability” we refer to what Hultsch et al. (2002) considered as “inconsistency within persons.” Specifically, in our study, we refer to the variability of a single measure evaluated across different trials within the same task.

In general, IIV changes on cognitive performance describe a U-shaped function during the lifespan (MacDonald et al., 2006), showing a quadratic relationship between IIV and age. At first, from childhood to adolescence, cognitive performance is characterized by a progressive reduction of IIV (Williams et al., 2005), followed by a relative stability during the early adulthood (Hultsch and MacDonald, 2012). Finally, with the advancing age in later adulthood, IIV encounters a further progressive increasing (Hultsch et al., 2002). In typically developing children, IIV decreases quickly after 6 years (Williams et al., 2005, 2007; Lewis et al., 2017), whereas compared to younger adults, older adults present more IIV (Hultsch et al., 2008; Garrett et al., 2012; Holtzer et al., 2014). Thus, IIV could be considered a stable and efficient marker of normal development and aging (MacDonald et al., 2006, 2009; Hultsch et al., 2008). Furthermore, a high fluctuation in response times, or response time variability, seems to reflect attentional lapses (Castellanos et al., 2005) and allows to acquire more information on short-term changes in behavior.

Altogether, these studies emphasize the fruitful “tool” of IIV in the study of cognitive functioning, without to assume a static vision of ontogenetic changes.

Intra-individual variability can be quantified in multiple ways and some measures typically used are the intra-individual standard deviation (ISD) and the coefficient of variation (CV). Alternatively, an interesting mathematical approach is the fitting of reaction time (RT) distributions through the use of mathematical functions. These methods allow to quantify different parameters of RT distributions, not only measures of central tendency such as the mean or median RTs.

For example, Ratcliff (1979) was among the first to show how the ex-Gaussian distribution yields an optimal fit to RT distributions. The ex-Gaussian is the convolution between a Gaussian and an exponential distribution and it can well capture the positive skew frequently observed in RT distributions (Luce, 2008). Three parameters can be estimated from the ex-Gaussian fitting. The *mu* (μ) and the *sigma* (σ) parameters represent the normally distributed components of the distribution. The *tau* (τ) represents the exponentially distributed component, which reflects the positive skew of the RT distribution, or in other words, the slowest RTs in a distribution.

Several authors provide cognitive interpretations attributed to the ex-Gaussian parameters. Specifically, the *tau* parameter would reflect a central processing component (Spieler et al., 1996), a measure of attentional lapses (Epstein et al., 2006), and an index of higher cognitive functions such as working memory and reasoning (Schmiedek et al., 2007). In general, the ex-Gaussian parameters seems to be an efficient method for measuring IIV (West et al., 2002; Tarantino et al., 2013; Lewis et al., 2017).

Interestingly, IIV across the lifespan would be associated with changes in brain morphology. The reduction of the gray matter during the development, or synaptic pruning, might contribute in the decrement of neural noise in cognitive functions and IIV (Gogtay et al., 2004). On the contrary, in the elderly population, a reduction of white matter, especially in the frontal lobes, may be related to the increased level of IIV (Moy et al., 2011).

Among cognitive processes that could better explain IIV across lifespan, executive functions are probably the best candidate for several reasons. First of all, neuroimaging studies provide evidence on a connection between executive processes mediated by frontal lobes and IIV (Bellgrove et al., 2004; Bunce et al., 2007). Second, executive functions are characterized by a multifaceted nature and it is plausible that a couple of its many components can be involved in IIV, namely lapses of intention (West et al., 2002) or an inadequate level of sustained attention (Kaiser et al., 2008).

In summary, although IIV has been linked to executive functions, so far no study has taken into account IIV of performance monitoring across the lifespan. This lack of evidence lays the foundations for investigating how EA and PES develop over the lifespan. Interestingly, IIV, considered from several authors a better predictor of normal development and aging than average performance (MacDonald et al., 2006, 2009; Hultsch et al., 2008), can offer new insights to characterize two phenomena closely related to performance monitoring, namely EA and PES.

## Materials and Methods

### Participants

Ninety-six healthy participants took part to the study: 34 children (age range 8–13 years), 30 younger adults (age range 19–35 years), and 32 older adults (age-range 61–83 years).

The inclusion criteria were normal or corrected-to-normal vision, no previous or present neurological and/or psychiatric disorders and no use of any neuro-psychopharmacological drugs. Older adults had an additional inclusion criterion of a Montreal Cognitive Assessment test score (Nasreddine et al., 2005) over the Italian cut-off (Conti et al., 2015; Santangelo et al., 2015). Moreover, participants with a poor or high level of accuracy on the EAT (Hester et al., 2005) were excluded from the analyses (<5% or >90% correctly withheld No-Go trials). As a result, the final sample consisted of 30 children (mean age 10.8 years, range 8–13), 30 younger adults (mean age 25.4 years, range 19–35), and 30 older adults (mean age 70.4 years, range 61–83). The **Table 1** shows the main demographics of the sample and the number of participants in each group entered in the analyses. Written informed consent was obtained from all of the younger and older participants, and from the parents of the children. The study was carried out in accordance with Declaration of Helsinki, and was approved by the Ethics Committee of School of Psychology, University of Padua.

**Table 1:** Participant demographics (overall sample) and number of participants in each group entered in the analyses.

Measure and analyses	Children	Younger adults	Older adults
Overall sample	*n* = 30	*n* = 30	*n* = 30
Mean age (*SD*)	10.8 (2)	25.4 (5)	70.4 (6)
Education	5 (1)	15 (3)	11.1 (6)
MoCA score (*SD*)	-	-	25.7 (2)
Average performance – RTs and accuracy	*n* = 30	*n* = 30	*n* = 30
Mean Error Awareness	*n* = 30	*n* = 30	*n* = 30
Error Awareness across time	*n* = 29	*n* = 28	*n* = 26
Intra-individual variability of Error Awareness	*n* = 29	*n* = 28	*n* = 26
Post-error slowing – means and SDs	*n* = 29	*n* = 29	*n* = 29
Post-error slowing – ex-Gaussian parameters	*n* = 29	*n* = 29	*n* = 29

*RTs, reaction times; SD, standard deviation; MoCA, Montreal Cognitive Assessment test.*

### Experimental Task

All participants performed a modified versions of the EAT, (Hester et al., 2005), a Go/No-Go response inhibition task specifically designed to evaluate EA. A serial stream of color words was presented at the center of a computer screen. The stimuli were presented at the center of a screen for 750 ms, followed by a black screen presented for 750 ms. Participants were asked to press a Go button (“3” on a keyboard), as soon as possible, when word and its font color were congruent (Go trial). On the contrary, participants had to withhold the response in two conditions: (1) when the same colored word was repeated on two consecutive trials (Repeat No-Go), or (2) when the word and its font color were incongruent (Stroop No-Go).

After each stimulus presentation a prompt was presented for 1000 ms, with the following question: “*Did you make a mistake*?”. During this time, participants were instructed to press an error button (space bar) to signal a supposed error. Through this prompt, the task was simplified compared to the original version (Hester et al., 2005), because participants were asked explicitly to monitor trial-by-trial own performance. In total 675 trials were presented (600 Go trials and 75 No-go trials, of which 36 Stroop No-Go and 39 Repeat No-Go). The task was divided into three blocks including 225 trials each, in order to guarantee participants took a brief rest after each block. It was ensured that all participants were well-trained and fully understood the instructions of the task before they began the experiment. The experiment was run by E-Prime software (version 2.0 Psychology Software Tools, Pittsburgh, PA, United States).

### Measures and Data Analysis

According to the aims of the present study, participant’s performance was evaluated through different dependent variables, which were calculated and analyzed as following.

#### Reaction Times and Accuracy

Average performance indices was assessed in term of mean RTs, for both correct and error responses (RTs under 100 ms were removed from analyses), and mean accuracy, which was calculated as the ratio of correct withholds on No-go trials.

To evaluate differences on mean RTs, a mixed 2 × 3 ANOVA was conducted with *response type* (correct vs. error) as a within-subjects variable and *group* (children, younger and older adults) as between-subjects variable. Differences in mean accuracy were computed by one-way ANOVA with *group* (children, younger, and older adults) as between-subjects variable.

#### Error Awareness

Mean EA was calculated as the percentage of correctly signaled errors on the total number of commission errors (O’Connell et al., 2009). Differences in mean EA were analyzed by one-way ANOVA with *group* (children, younger, and older adults) as between-subjects variable. To evaluate EA across the time (task duration), we divided the EAT into six blocks and computed EA for each of them. In this case, a mixed 6 × 3 ANOVA was conducted with *block* (1, 2, 3, 4, 5, and 6) as within-subjects variable and *group* (children, younger, and older adults) as between-subjects variable. For this analysis, the sample size was reduced to 83 participants (29 children, 28 younger, and 26 older adults) because seven of them did not commit any error during a particular block (see **Table 1**).

Finally, in order to evaluate IIV of EA across the task duration, a single value of SD was computed for each participant, again considering EA across the six blocks. Standard deviations were averaged for each group and compared by one-way ANOVA with *group* (children, younger, and older adults) as between-subjects variable. Again, for these analyses the sample size was reduced to 83 participants (29 children, 28 younger, and 26 older adults) (see **Table 1**).

#### Post-error Slowing

Mean PES was computed according to Dutilh et al. (2012) by the difference between the RT that follows and precedes each error. This difference was compared with the difference between the RT that follows and precedes each correct withhold. RTs under 100 ms were removed from analyses. Unaware errors were excluded from the analyses of PES. Moreover, three participants were removed from the analyses since did not commit at least 3 errors to compute a reliable mean PES (Fitzgerald et al., 2010; Rodehacke et al., 2014). Consequently, the sample size was reduced to 87 participants (29 children, 29 younger, and 29 older adults) for all the analyses on PES (see **Table 1**). The outline of the analyses on PES is as follows. First, differences in terms of mean and SD were analyzed by two mixed 2 × 2 × 3 ANOVA with *response* (post vs. pre No-Go target response) and *target response* (aware error vs. correct withhold) as within-subjects variables and *group* (children, younger, and older adults) as between-subjects variable. Finally, as measures of IIV of PES, we fitted the ex-Gaussian distribution to our RT distributions. The ex-Gaussian parameters were calculated by the *egfit* MATLAB function (Lacouture and Cousineau, 2008) that allows estimating the measure of central tendency (*mu*), spread (*sigma*), and the degree of positive skew (*tau*) of the distribution. Three separated mixed 2 × 2 × 3 ANOVAs were conducted for each ex-Gaussian parameter, in which *response* (post vs. pre No-Go target response) and *target response* (aware error vs. correct withhold) were entered as within-subjects variables and *group* (children, younger, and older adults) as between subject variables.

We decided to avoid investigating PES across the time (task duration), because a sufficient number of trials was not present in all the conditions of a hypothetical 6 × 2 × 2 × 3 ANOVA with *block* (1, 2, 3, 4, 5, 6), *response* (post vs. pre No-Go target response) and *target response* (aware error vs. correct withhold) as within-subjects variables and *group* (children, younger, and older adults) as between-subjects variable.

In general, the Bonferroni correction was applied to every *post hoc* analysis and a corrected alpha-level of 0.05 was considered. Finally, effect sizes were estimated by partial eta squared (${\text{η}}_{\text{p}}^{2}$).

## Results

### Reaction Times and Accuracy

The mean and SD of RTs and accuracy are showed in **Table 2**. Data analyses on mean RTs revealed a main effect of *response type* [*F*(1,87) = 32.4, *p* < 0.001, ${\text{η}}_{\text{p}}^{2}$ = 0.3], and *group* [*F*(2,87) = 21.8, *p* < 0.001, ${\text{η}}_{\text{p}}^{2}$ = 0.3]. *Post hoc* comparisons showed that error RTs were faster than correct RTs. Moreover, younger adults were faster than children (*p* < 0.01) and older adults (*p* < 0.001), whereas children were faster than older adults (*p* < 0.05). No interaction between *response type* × *group* was found.

**Table 2:** Mean and standard deviations (*SD*) of performance indices on the EAT for children, younger, and older adults.

	Children	Younger adults	Older adults
	Mean (*SD*)	Mean (*SD*)	Mean (*SD*)
Correct RT (ms)	572 (103)	484 (60)	636 (96)
Error RT (ms)	544 (121)	464 (63)	613 (88)
Accuracy (%)	28 (20)	52 (22)	60 (18)
Mean error awareness (%)	65 (18)	89 (8)	56 (19)
Error awareness – block 1 (%)	48 (33)	85 (18)	52 (32)
Error awareness – block 2 (%)	66 (27)	88 (14)	51 (28)
Error awareness – block 3 (%)	68 (24)	86 (14)	61 (31)
Error awareness – block 4 (%)	68 (22)	90 (14)	58 (32)
Error awareness – block 5 (%)	76 (18)	89 (20)	64 (33)
Error awareness – block 6 (%)	62 (25)	91 (12)	56 (37)

As for mean accuracy, a main effect of *group* was found [*F*(2,87) = 20.8, *p* < 0.001, ${\text{η}}_{\text{p}}^{2}$ = 0.3]. *Post hoc* comparisons showed that children made more errors than younger adults (*p* < 0.001) and older adults (*p* < 0.001). No difference between younger and older adults in terms of accuracy (*p* = 0.32).

### Error Awareness

The mean and SD of EA scores are presented in **Table 2**. The analyses on mean EA revealed a main effect of *group* [*F*(2,87) = 33.03, *p* < 0.001, ${\text{η}}_{\text{p}}^{2}$ = 0.4]. The *post hoc* comparisons indicated that younger adults were more aware on their commission errors respect to both the children (*p* < 0.001) and older adults (*p* < 0.001). No significant differences were found comparing mean EA in children and older adults (*p* = 0.14).

**FIGURE 1:**
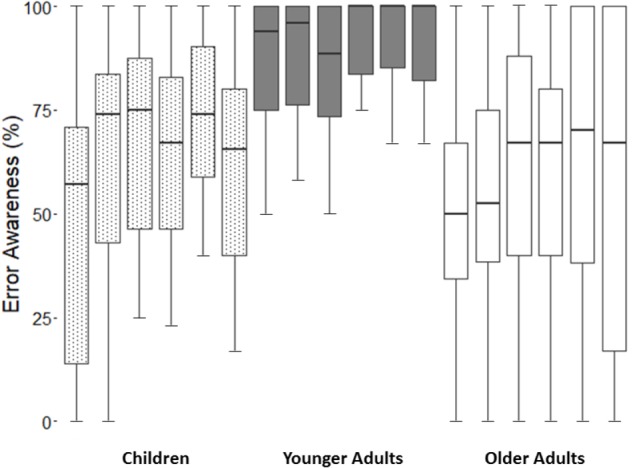
The figure shows block-by-block error awareness scores for each group. From left to right: Children-Block 1, 2, 3, 4, 5, 6; Younger Adults-Block 1, 2, 3, 4, 5, 6; Older Adults-Block 1, 2, 3, 4, 5, 6.

When EA across the time were considered in our analyses (**Figure 1**), we found a main effect of *block* [*F*(4.1,329.5) = 4.6, *p* < 0.05, ${\text{η}}_{\text{p}}^{2}$ = 0.1]. Specifically, *post hoc* comparisons showed a general improvement in EA between the block 1 and 5 (*p* < 0.01). However, this improvement disappeared at the end of the task, as the comparison between the blocks 1 and 6 showed (*p* = 0.29). Furthermore, the analyses revealed a main effect of *group* [*F*(2,80) = 26.5, *p* < 0.001, ${\text{η}}_{\text{p}}^{2}$ = 0.4]. Similarly to the analyses on mean EA, we observed by *post hoc* comparisons that younger adults had a higher level of EA than children (*p* < 0.001) and older adults (*p* < 0.001). The comparison between children and older adults did not show a significant difference (*p* = 0.29). Finally, no interaction between *block* × *group* was observed (*p* = 1.57).

The analyses of IIV of EA across time, in terms of SD, showed a main effect of *group* [*F*(2,80) = 15.1, *p* < 0.001, ${\text{η}}_{\text{p}}^{2}$ = 0.3]. Younger adults’ EA was less variable than children (mean standard deviations: 12 vs. 19; *p* < 0.05) and older adults (mean standard deviations: 12 vs. 26; *p* < 0.001). Interestingly, unlike mean EA that did not reveal a difference between children and older adults in terms of average performance, the comparison of standard deviations showed that children had a lower IIV of EA than older adults (mean standard deviations: 19 vs. 26; *p* < 0.05).

### Post-error Slowing – Means and SDs

**FIGURE 2:**
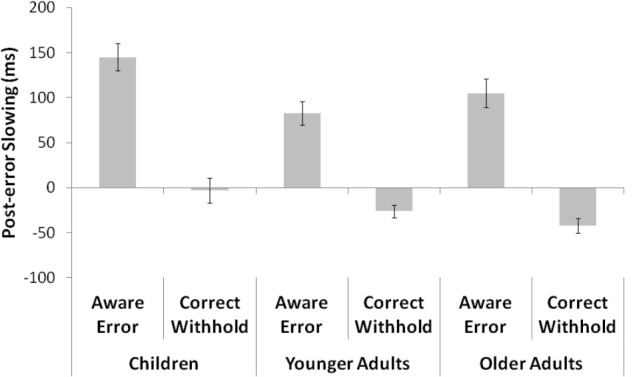
The figure shows the difference between RTs following and prior an aware error or a correct withhold for each group. Post-error slowing is clearly visible after an aware error.

The mean and SD for RTs following and prior an aware error or a correct withhold are showed in **Table 3**. The analyses on means showed a main effect of *response* [*F*(1,84) = 78.53, *p* < 0.001, ${\text{η}}_{\text{p}}^{2}$ = 0.5], *target response* [*F*(1,84) = 25.73, *p* < 0.001, ${\text{η}}_{\text{p}}^{2}$ = 0.2], and *group* [*F*(2,84) = 21.66, *p* < 0.001, ${\text{η}}_{\text{p}}^{2}$ = 0.3]. The *post hoc* comparisons indicated that RTs following a No-Go target were slower than RTs prior a No-Go target (*p* < 0.001), and RTs faster (without a distinction between post and pre No-Go target response) when participants correctly withheld the response for No-Go target (*p* < 0.001). As for group differences, younger adults were generally faster than children (*p* < 0.01) and older adults (*p* < 0.001), whereas children and older adults did not present any difference (*p* = 0.32). Moreover, the analyses revealed a *response* × *group* interaction [*F*(2,84) = 8.1, *p* < 0.01, ${\text{η}}_{\text{p}}^{2}$ = 0.2]. All groups showed differences each other (all *p* < 0.01), namely a slowing after a No-Go target. Finally, an interaction between *response* × *target response* was found [*F*(2,84) = 8.1, *p* < 0.01, ${\text{η}}_{\text{p}}^{2}$ = 0.2], confirming a PES effect (**Figure 2**). In fact, data revealed a general slowing after an aware error (post-error RTs = 650 ms vs. pre-error RTs = 539 ms; *p* < 0.001) and an opposite trend after a correct withhold (post-withhold RTs = 550 ms vs. pre-withhold RTs = 573 ms; *p* < 0.001). No *response* × *target response* × *group* interaction was found (*p* = 0.24), revealing that the magnitude of PES was the same in all groups.

**Table 3:** Means and standard deviations (*SDs*) for post- and pre-target responses computed as a function of target response (aware error, correct withhold) and group.

		Children	Younger adults	Older adults
		Mean (*SD*)	Mean (*SD*)	Mean (*SD*)
Means	Post-target (aware error)	684 (136)	551 (101)	716 (117)
	Pre-target (aware error)	538 (90)	468 (52)	610 (98)
	Post-target (correct withhold)	590 (109)	470 (50)	590 (95)
	Pre-target (correct withhold)	591 (117)	496 (51)	631 (91)
Standard deviations	Post-target (aware error)	175 (61)	117 (48)	143 (59)
	Pre-target (aware error)	155 (46)	88 (22)	97 (55)
	Post-target (correct withhold)	133 (55)	88 (33)	97 (53)
	Pre-target (correct withhold)	161 (65)	95 (26)	100 (46)

The analyses on SDs revealed a main effect of *response* [*F*(1,84) = 4.94, *p* < 0.05, ${\text{η}}_{\text{p}}^{2}$ = 0.1], *target response* [*F*(1,84) = 12.76, *p* < 0.01, ${\text{η}}_{\text{p}}^{2}$ = 0.1], and *group* [*F*(2,84) = 24.1, *p* < 0.001, ${\text{η}}_{\text{p}}^{2}$ = 0.4]. The *post hoc* comparisons indicated that RTs following a No-Go target were more variable than RTs prior a No-Go target (*p* < 0.05), and a reduction of variance (collapsing post and pre No-Go target response) when participants correctly withheld the response for No-Go target (*p* < 0.05). The main effect on *group* indicated that children’ RTs were generally more inconsistent than younger adults (*p* < 0.001), and older adults (*p* < 0.001). No difference between younger and older adults (*p* = 0.53). Moreover, the analyses on SDs revealed a *response* × *target response* interaction [*F*(1,84) = 27.9, *p* < 0.001, ${\text{η}}_{\text{p}}^{2}$ = 0.2]. The results showed a general increasing of variance after an aware error (post-error *SDs* = 145 vs. pre-error *SDs* = 113; *p* < 0.001) and an opposite trend after a correct withhold (post-withhold *SDs* = 106 vs. pre-withhold *SDs* = 118; *p* < 0.05). No interaction between *response* × *target response* × *group* was found (*p* = 0.76).

### Post-error Slowing – Ex-Gaussian Parameters

The **Table 4** summarizes the results (means and SDs) on ex-Gaussian parameters. Three mixed ANOVAs were conducted to check differences on each parameter of the distribution: *mu, sigma*, and *tau.*

**Table 4:** Ex-Gaussian parameters for post- and pre-target responses computed as a function of target response (aware error, correct withhold) and group.

		Children	Younger adults	Older adults
		Mean (*SD*)	Mean (*SD*)	Mean (*SD*)
*Mu*	Post-target (aware error)	532 (115)	457 (89)	612 (118)
	Pre-target (aware error)	418 (98)	402 (68)	559 (96)
	Post-target (correct withhold)	509 (127)	418 (29)	519 (88)
	Pre-target (correct withhold)	498 (125)	421 (59)	552 (83)
*Sigma*	Post-target (aware error)	63 (52)	50 (31)	60 (44)
	Pre-target (aware error)	72 (58)	43 (24)	56 (40)
	Post-target (correct withhold)	79 (54)	56 (28)	47 (22)
	Pre-target (correct withhold)	84 (71)	46 (25)	44 (17)
*Tau*	Post-target (aware error)	152 (91)	94 (57)	104 (77)
	Pre-target (aware error)	122 (60)	66 (35)	51 (50)
	Post-target (correct withhold)	82 (64)	52 (36)	71 (60)
	Pre-target (correct withhold)	93 (71)	75 (35)	80 (51)

The analyses on the *mu* parameter showed a main effect of *response* [*F*(1,84) = 30.9, *p* < 0.001, ${\text{η}}_{\text{p}}^{2}$ = 0.3] and *group* [*F*(2,84) = 20.96, *p* < 0.001, ${\text{η}}_{\text{p}}^{2}$ = 0.3]. Similarly to the previous analyses on means, RTs following a No-Go target were slower than RTs prior a No-Go target (*p* < 0.001). The main effect on *group* revealed that younger adults were generally faster than children (*p* < 0.01) and older adults (*p* < 0.001), and children were faster than older adults (*p* < 0.01). In addition, the analyses revealed a *response* × *group* interaction [*F*(2,84) = 6.8, *p* < 0.01, ${\text{η}}_{\text{p}}^{2}$ = 0.1]. Only younger adults and children presented a slowing after a No-Go target (*p* < 0.05). Interestingly, an interaction between *response* × *target response* was found [*F*(1,84) = 46.1, *p* < 0.001, ${\text{η}}_{\text{p}}^{2}$ = 0.4], confirming PES also when the *mu* parameter was entered in the analyses, rather than the more conventional mean. The results showed higher *mu* scores after an aware error (post-error *mu* = 534 vs. pre-error *mu* = 460; *p* < 0.001), whereas no difference after a correct withhold (post-withhold *mu* = 482 vs. pre-withhold *mu* = 490; *p* = 0.32). Finally, in line with the previous analyses, no *response* × *target response* × *group* interaction was found (*p* = 0.30).

The analyses on the *sigma* parameter showed only a main effect of *group* [*F*(2,84) = 9, *p* < 0.001, ${\text{η}}_{\text{p}}^{2}$ = 0.2]. *Post hoc* analyses revealed that children showed higher scores of *sigma* than younger adults (*p* < 0.01) and older adults (*p* < 0.01). No differences in terms of *sigma* scores between younger and older adults (*p* = 1).

Finally, the analyses on the *tau* parameter showed a main effect of *response* [*F*(1,84) = 4, *p* < 0.05, ${\text{η}}_{\text{p}}^{2}$ = 0.05], *target response* [*F*(1,84) = 14.57, *p* < 0.001, ${\text{η}}_{\text{p}}^{2}$ = 0.1], and *group* [*F*(2,84) = 11.35, *p* < 0.001, ${\text{η}}_{\text{p}}^{2}$ = 0.2]. The *post hoc* comparisons indicated higher *tau* scores after a No-Go target than *tau* scores preceding a No-Go target (*p* < 0.05), and lower *tau* scores (without a distinction between post and pre No-Go target response) when participants correctly withheld the response for No-Go target (*p* < 0.001). As for group differences, children had higher *tau* scores than younger adults (*p* < 0.001) and older adults (*p* < 0.01). No difference between younger and older adults (*p* = 1). Finally, an interaction between *response* × *target response* was found [*F*(1,84) = 17.21, *p* < 0.001, ${\text{η}}_{\text{p}}^{2}$ = 0.2]. The results showed higher *tau* scores after an aware error (post-error *tau* = 117 vs. pre-error *tau* = 80; *p* < 0.001), whereas no difference after a correct withhold (post-withhold *tau* = 68 vs. pre-withhold *tau* = 82; *p* = 0.06). No *response* × *target response* × *group* interaction was found (*p* = 0.78).

## Discussion

The purpose of the present study was to investigate the ontogenetic trajectories of EA and PES through the comparison of different age-groups and how EA and PES interact with each other. The performance of children (age range 8–13 years), younger adults (age range 19–35 years) and older adults (age range 61–83 years) in a modified version of the EAT (Hester et al., 2005) was compared. In particular, in this study not only variations of the average performance were examined, but also IIV, considered in terms of variations of SD and ex-Gaussian parameters (*mu*, *sigma*, and *tau*).

The results on average performance on the EAT showed that older adults were generally slower than younger adults and children. However, in terms of accuracy, older and younger adults did not differ. This result is in line with several studies in which a general slower performance in older adults, but same levels of accuracy than younger adults, seems partially to reflect a changing in the strategy used to tackle a task, indeed older adults seem to be more caution that younger adults in their responses (Starns and Ratcliff, 2010; Dutilh et al., 2013). In contrast, accuracy in children was lower than the other two groups, since they committed more errors, showing a difficulty to inhibit an inappropriate response and in general a lower level of inhibitory control.

Particularly interesting is the result in which EA was more reduced in children and older adults, rather than in younger adults. Previous studies have already highlighted differences between older and younger adults, in terms of EA, showing a poorer levels of error detection in older adults (Harty et al., 2013, 2014, 2017). However, for the first time, this study also takes into account a group of children, in order to outline the ontogenetic trajectory of EA comparing different age-groups. The results showed that EA in children was not yet a fully mature process if compared to a group of younger adults. At the same time, the results confirmed the previous evidence that EA is reduced in older adults. Taken together, these findings show a pretty clear relationship between age and EA. Specifically, these results suggest that the relationship between age and EA could be represented by a classical U-shaped function, as it is for most of the executive functions. Whereas EA increases through childhood and early adulthood, advancing age in late adulthood is related to a reduction of EA. However, the absence of a fourth group of adults aged between 40 and 60, which is a limitation of this study, suggests waiting for future replication in order to confirm this conclusion.

When we evaluated EA across the time, dividing the task into six blocks, we found an improvement in EA between the blocks 1 and 5 and, afterward, a reduction in EA at the end of the task, as the comparison between the blocks 1 and 6 showed. This result could depend on a spontaneous fluctuation in sustained attention or arousal during the EAT and it seems to be in line with previous studies that show a relationship between EA and arousal (Shalgi et al., 2007; Robertson, 2014).

Of interest, IIV of EA across the time showed a different pattern, because it was more prominent in older than younger adults and children. Although older adults and children, in terms of average performance, had very similar levels of EA, older adults were characterized by a more marked IIV of EA across the time. A possible interpretation of these results could derive from previous studies where an increased IIV has been often associated with impairments in attention (Castellanos et al., 2005), especially in older adults (Hultsch et al., 2002). Thus, temporary lapses of attention in older adults can contribute to explain a greater inconsistency of EA.

The results of our study revealed PES, a phenomenon widely observed in the literature. However, the strength of the present study is that PES was examined using both more conventional approach based on the comparison of the mean and SD for RTs, and the use of the ex-Gaussian function to fit our RT distributions.

The first evidence that emerges from our findings concerns the fact that PES was confirmed, as expected when the mean RTs were considered. Moreover, interestingly, we also observed PES when the *mu* parameter of the ex-Gaussian was entered in the analyses.

As for the absence of an interaction between *response* × *target response* × *group*, when both mean RTs and *mu* parameter were considered in the analyses, it revealed no difference between groups in terms of the magnitude of PES. This result contrasts our expectations and also previous findings that showed a more pronounced PES in older adults (Dutilh et al., 2013). In fact, we expected to find group differences regarding the magnitude of PES, in particular, we expected these differences were greater in children and older adults, compared to younger adults. These expectations were justified by at least two different interpretations. On the one hand, we expected an increased PES in children and older adults since it is generally known they present greater difficulty in inhibitory control (Christ et al., 2001). As claimed by Notebaert et al. (2009), PES would reflect a slow down generated by an orientating response when an error occurs. The easier distractibility in children and older adults to exogenous and endogenous stimuli (e.g., an error that represents a failure in performance monitoring) could, therefore, explain the greater expected slow down. On the other hand, especially to explain the expected slowing in older adults, it is known they seem to be more cautious to avoid errors, as well described by the speed-accuracy trade off phenomenon. Therefore, it was plausible to expect a major slow down following an error in older adults. In summary, both weak inhibitory control and changes in the strategy used to tackle a task could explain our expectations. However, the unexpected result, in the present study, opens up an alternative interpretation. In fact, considering PES as a compensatory and adaptive process aimed at improving performance following an error (Gehring and Fencsik, 2001), it is plausible to think that it is already well mature in childhood and do not decline during normal aging. This interpretation would explain the absence of differences between the groups in terms of the magnitude of PES.

Another interesting result concerns the *response* × *target response* interaction found when we considered into the analyses on PES the SD of the RT distributions. This interaction showed that people not only slow down on trials following errors, but also are more inconsistent in following responses.

The last group of analyses took the three parameters of the ex-Gaussian distribution into account. The results revealed differences between the three groups in terms of the normally distributed RT *mu* parameter. In fact, older adults were the slowest group, while children were slower than younger adults. This result differed from the previous analyses of mean RTs of PES, since in that analyses no difference was present between children and older adults. Therefore, excluding the slowest RTs from the distributions (in fact the slowest RTs of the distribution were captured by the *tau* parameter), the group of children appeared to be faster than older adults.

When we considered the *sigma* parameter, we found a main effect of group. The results showed that the normally distributed RTs of the ex-Gaussian were more variable in children than younger and older adults. These results were perfectly consistent to those observed when the SDs were considered in the analyses of PES.

Finally, the analyses on the *tau* parameter revealed an important result. Children presented the highest *tau* scores compared to younger and older adults. In previous studies, *tau* values have been associated with a higher IIV (see Dixon et al., 2004) and this inconsistency has been related to lapses in cognitive processes, such as attention. In particular, high *tau* scores would be related to attentional lapses (Epstein et al., 2006). This evidence can partially explain the differences between groups observed in our study regarding *tau* scores. Children may be more prone to attentional lapses than the other two groups and consequently to present a higher level of IIV.

## Conclusion

This study allows observing two distinct ontogenetic trajectories of the investigated processes. Regarding EA, we suggest the existence of a U-shaped curve that describes an increase of the process from childhood to early adulthood and a progressive reduction advancing age in late adulthood. Furthermore, a greater IIV in older adults indicated a susceptibility of EA to the aging process. The ontogenetic trajectory of PES seems substantially different from the trajectory that describes EA since in PES we do not observe age-related differences. These results suggest that EA and PES are two independent processes, explaining why in a previous study no association between them was revealed (Cohen, 2009). Furthermore, it appears that EA and PES are differently prone to short-term fluctuations in performance across the lifespan. While EA presents an increase in IIV in aging, PES seems to be immune to these changes.

## Author Contributions

FM, ED, and DM conceived and planned the experiments. FM and ED carried out the experiments. FM analyzed the data and wrote the manuscript with input from all authors. ED and DM contributed to the interpretation of the results.

## Conflict of Interest Statement

The authors declare that the research was conducted in the absence of any commercial or financial relationships that could be construed as a potential conflict of interest.
